# Lessons for livestock genomics from genome and transcriptome sequencing in cattle and other mammals

**DOI:** 10.1186/s12711-016-0237-6

**Published:** 2016-08-17

**Authors:** Jeremy F. Taylor, Lynsey K. Whitacre, Jesse L. Hoff, Polyana C. Tizioto, JaeWoo Kim, Jared E. Decker, Robert D. Schnabel

**Affiliations:** 1 Division of Animal Sciences, University of Missouri, Columbia, MO USA; 2 Informatics Institute, University of Missouri, Columbia, MO USA; 3 Embrapa Southeast Livestock, São Carlos, SP Brazil

## Abstract

**Background:**

Decreasing sequencing costs and development of new protocols for characterizing global methylation, gene expression patterns and regulatory regions have stimulated the generation of large livestock datasets. Here, we discuss experiences in the analysis of whole-genome and transcriptome sequence data.

**Methods:**

We analyzed whole-genome sequence (WGS) data from 132 individuals from five canid species (*Canis familiaris*, *C. latrans*, *C. dingo*, *C. aureus* and *C. lupus*) and 61 breeds, three bison (*Bison bison*), 64 water buffalo (*Bubalus bubalis*) and 297 bovines from 17 breeds. By individual, data vary in extent of reference genome depth of coverage from 4.9X to 64.0X. We have also analyzed RNA-seq data for 580 samples representing 159 *Bos taurus* and *Rattus norvegicus* animals and 98 tissues. By aligning reads to a reference assembly and calling variants, we assessed effects of average depth of coverage on the actual coverage and on the number of called variants. We examined the identity of unmapped reads by assembling them and querying produced contigs against the non-redundant nucleic acids database. By imputing high-density single nucleotide polymorphism data on 4010 US registered Angus animals to WGS using Run4 of the 1000 Bull Genomes Project and assessing the accuracy of imputation, we identified misassembled reference sequence regions.

**Results:**

We estimate that a 24X depth of coverage is required to achieve 99.5 % coverage of the reference assembly and identify 95 % of the variants within an individual’s genome. Genomes sequenced to low average coverage (e.g., <10X) may fail to cover 10 % of the reference genome and identify <75 % of variants. About 10 % of genomic DNA or transcriptome sequence reads fail to align to the reference assembly. These reads include loci missing from the reference assembly and misassembled genes and interesting symbionts, commensal and pathogenic organisms.

**Conclusions:**

Assembly errors and a lack of annotation of functional elements significantly limit the utility of the current draft livestock reference assemblies. The Functional Annotation of Animal Genomes initiative seeks to annotate functional elements, while a 70X Pac-Bio assembly for cow is underway and may result in a significantly improved reference assembly.

**Electronic supplementary material:**

The online version of this article (doi:10.1186/s12711-016-0237-6) contains supplementary material, which is available to authorized users.

## Background

This paper serves to report on a presentation that was made at the International Symposium on Animal Functional Genomics conference in Piacenza, Italy that was held between July 27 and 29, 2015. Consequently, some of the material reported here has been published elsewhere [1–4] and is appropriately cited, while the remainder has not been previously published. Our objective is to present a synthesis of some of the more important findings that we have arrived at after several years of analyzing whole-genome sequences from dogs, cattle and bison and whole-transcriptome data from a variety of tissues derived from rat and cattle. In particular, we focus on the limitations of the draft reference genome assemblies for livestock species by analyzing the errors that are inherent to the current UMD3.1 bovine assembly. While it is imperative that reference genome assemblies be improved for their sequence content, their information content must also be dramatically improved through their functional annotation.

The first genome that our group sequenced in May 2010 was for a Basenji dog with Fanconi syndrome. Fanconi syndrome is an autosomal recessive adult onset disorder with generalized proximal tubule reabsorption deficiency that manifests as renal failure and is invariably lethal. We had conducted a pedigree-based linkage analysis using microsatellite loci in an extended family of dogs and localized the causal variant to a region between 35 and 50 Mb on chromosome 3. Fine-mapping of this region led to the identification of a core haplotype that spanned 2.7 Mb and contained 27 genes, none of which were obvious candidates for the disease. All affected dogs were homozygous for this haplotype. Thus, our next step would have been to individually amplify by polymerase chain reaction and sequence all the exons of these 27 genes to attempt to identify the causal mutation. However, since the University of Missouri had just purchased a new Illumina Genome Analyzer, an alternative and seemingly much simpler strategy, at least for the generation of data, was to sequence the entire genome of an affected dog and restrict our analysis to only the 0.1 % of the generated data that corresponded to the critical region that was predicted to harbor the causal variant. An obvious advantage of this strategy was that sequence data would be generated for the entire region and thus if the causal variant happened to lie outside of an exon, it would be captured. What was less obvious to us at the time was the fact that although the causal mutation was almost certainly included in the generated data, our ability to detect it was by no means guaranteed. Our experience of such analyses at the time was limited to the discovery of variants that we had conducted for the design of the Illumina BovineSNP50 assay and this involved alignment of the sequences against a reference genome and calling variants. In other words, our experience and the capability of most of the available software programs at the time were limited to identifying variants that were simple to detect and common in the genome. With time, we came to understand that: (1) assembly errors including missing sequences significantly impact the discovery of variants; (2) variant callers fail to identify large homozygous deletions since where there is no sequence to align, there are no variants to call; (3) large insertions in re-sequenced genomes will not align to the reference genome and will probably not be identified if the unmapped reads are not carefully examined; (4) depth of sequence coverage is important for genotyping-by-sequencing both from the perspective of the discovery of heterozygous variants and the proportion of the genome that is covered by reads; and (5) large deletions and duplications are difficult to detect as well as other classes of copy number variants. In this paper, we discuss some of our experiences in tackling these issues in the analysis of RNA-seq and whole-genome sequence data.

## Methods

### Ethics statement

All tissue sampling that was performed for the analyses carried out for this work were performed under protocol 7505 approved by the University of Missouri Animal Care and Use Committee.

### Animal sampling and sequencing

The animals sampled for this study were sequenced for a variety of reasons and for a number of different projects. The 132 sequenced canid individuals include coyote, dingo, jackal, wolf [5] and 61 dog breeds (Table 1). The sequenced wild dog species were provided by collaborators at the University of California, Los Angeles, who have jointly analyzed wild and domesticated dog breeds’ sequences to examine the effects of domestication and breed formation on the shaping of the distribution of deleterious variation within the genome [1]. The breed dogs were primarily sequenced at the University of Missouri (MU) or provided by collaborators to identify variants that cause inherited neurological diseases [6–13]. Our current strategy is to deep sequence the genomes of affected dogs only that are presumed to be homozygous for a recessive Mendelian variant that disrupts the function of an unknown gene, and then to identify all the candidate variants within genes for which an affected dog is homozygous and the unaffected dogs of other breeds are homozygous for the reference allele. The wild dogs were individually sequenced to an average depth of 6.1–60.6X (25.4 ± 13.4X) while the breed dogs were individually sequenced to an average depth of 6.3–38.0X (22.0 ± 7.3X). DNA was extracted from white blood cells.

**Table 1:** Illumina whole-genome sequence data analyzed for canids

Breed or species^a^	Number of animals	Number of unique reads	Total number of bases	Average raw coverage
Airedale	3	1,835,972,548	189,076,779,986	21.73
American Cocker Spaniel	1	550,728,624	49,677,215,966	17.13
Australian Cattle Dog	1	711,273,698	70,765,609,261	24.40
Australian Shepherd	2	840,566,934	85,850,839,503	14.80
Basenji	4	2,927,119,952	210,417,110,609	18.14
Beagle	1	780,817,394	63,747,724,135	21.98
Berger Picard	3	2,069,275,590	205,948,372,326	23.67
Black and Tan Coonhound	1	679,891,938	67,638,413,640	23.32
Black Russian Terrier	1	997,183,630	99,214,491,903	34.21
Border Collie	4	2,060,945,654	198,204,250,786	17.09
Border Terrier	3	2,176,208,598	216,516,863,418	24.89
Boxer	1	697,830,122	69,426,054,504	23.94
Brittany Spaniel	1	270,637,588	24,978,627,038	8.61
Cane Corso	1	739,372,554	73,535,208,078	25.36
Cavalier King Charles Spaniel	1	265,927,732	28,587,068,953	9.86
Chinese Crested	2	1,438,761,694	146,160,620,792	25.20
Chinook	1	504,935,430	49,629,756,311	17.11
Clumber Spaniel	1	433,000,560	46,550,630,335	16.05
Doberman Pinscher	3	2,344,622,200	238,904,843,602	27.51
Dogue de Bordeaux	1	170,689,396	18,347,702,387	6.33
English Cocker Spaniel	2	1,281,420,632	126,908,789,998	21.88
English Pointer	1	567,753,520	55,967,932,647	19.30
English Setter	1	634,717,832	63,149,492,667	21.78
English Springer Spaniel	2	1,590,523,446	158,194,631,252	27.27
German Shepherd	1	756,040,450	75,156,450,593	25.92
Golden Retriever	5	4,070,501,036	403,283,810,728	27.81
Gordon Setter	1	817,775,346	81,358,201,786	28.05
Great Dane	1	376,806,522	37,839,711,742	13.05
Great Pyrenees	1	565,784,774	49,568,606,807	17.09
Irish Setter	1	346,307,834	34,795,164,362	12.00
Italian Greyhound	2	1,542,147,642	149,486,662,073	25.77
Jack Russell Terrier	4	3,105,361,266	308,784,422,151	26.62
Kangal	1	684,150,446	67,965,611,329	23.44
Kerry Blue Terrier	1	687,611,948	68,389,683,123	23.58
Kerry Blue Terrier x Beagle	1	651,841,508	64,849,405,821	22.36
Labrador Retriever	5	3,709,095,580	353,808,502,687	24.40
Lowchen	1	715,653,408	71,198,644,281	24.55
Mastiff	1	483,681,504	48,119,364,526	16.59
Minature Schnauzer	1	195,414,668	18,621,971,238	6.42
Newfoundland	1	823,220,602	81,614,127,419	28.14
Norwegian Lundehund	1	811,706,582	68,482,430,848	23.61
Nova Scotia Duck Tolling Retriever	1	596,223,330	59,315,213,926	20.45
Pembroke Welsh Corgi	3	2,051,796,660	203,917,418,863	23.44
Pointer	1	679,321,384	67,449,360,020	23.26
Portuguese Podengo	1	713,936,158	70,998,114,063	24.48
Portuguese Pointer	1	248,203,298	26,681,270,396	9.20
Pug	5	2,181,139,846	211,948,096,064	14.62
Racing Greyhound	1	506,353,446	44,692,996,771	15.41
Rhodesian Ridgeback	1	675,657,188	57,628,159,808	19.87
Rottweiler	2	1,725,459,344	171,619,235,087	29.59
Saint Bernard	1	686,645,884	68,287,808,505	23.55
Saluki	1	511,267,106	54,961,103,147	18.95
Scottish Deerhound	1	621,980,494	61,860,209,481	21.33
Scottish Terrier	2	1,885,266,610	187,558,882,079	32.34
Shetland Sheepdog	2	1,234,686,882	136,146,946,574	23.47
Shiba Inu	1	630,025,762	62,680,763,830	21.61
Soft Coated Wheaten Terrier	4	2,032,830,308	202,332,623,664	17.44
Standard Poodle	4	2,327,588,144	228,963,620,584	19.74
Standard Schnauzer	1	747,864,414	74,406,225,393	25.66
Tibetan Terrier	2	1,269,955,028	126,330,578,452	21.78
West Highland White Terrier	5	4,316,424,718	430,004,352,287	29.66
*Canis aureus* (Jackal)	3	8,351,392,646	830,865,082,100	30.48
*Canis dingo* (Dingo)	1	3,042,961,175	302,630,047,893	50.02
*Canis latrans* (Coyote)	2	9,521,271,994	942,508,023,731	17.25
*Canis lupus* (Wolf)	15	96,911,894,312	8,634,051,009,336	23.80

^a^Many of these sequences were used in the study by Marsden et al. [1]

The 364 sequenced bovid individuals include: three North American bison (*Bison bison*) sequenced at MU, 64 water buffalo (*Bubalus bubalis*) sequenced by members of the International Water Buffalo Genome Project and 297 sequenced cattle that represented 17 *Bos taurus taurus* and *B. taurus indicus* breeds (Table 2). Cattle were individually sequenced by collaborators at the USDA Beltsville Agricultural Research Center for a study on copy number variation [14, 15], at the University of Alberta as part of the 1000 Bull Genomes Project [16], and at MU for variant detection [2, 17] and to enable the design of the Neogen GGP-F250 assay, an Illumina 250K BeadChip, which primarily contains single nucleotide polymorphisms (SNPs) that are predicted to be functional based on their potential to affect gene products (unpublished data). Data for the reference animal, i.e., L1 Dominette 01449, were generated at the USDA Beltsville Agricultural Research Center, the University of California, Davis and by BGI (Shenzhen, China). The three bison individuals were individually sequenced to an average depth of 30.4–41.4X (34.8 ± 5.8X) while the 64 water buffalo animals were individually sequenced to an average depth of 1.1–12.3X (5.1 ± 3.4X). In both cases, the UMD3.1 bovine reference assembly was used. The taurine cattle were individually sequenced to an average depth of 0.3–64.0X (19.8 ± 11.3X) and the indicine and taurine × indicine hybrid cattle were sequenced to an average depth of 0.9–32.0X (13.0 ± 10.7X). DNA was extracted from semen or white blood cells except for L1 Dominette 01449 for which DNA was extracted separately from white blood cells and also from a liver sample.

**Table 2:** Illumina whole-genome sequence data analyzed for bovids

Breed or species	Number of animals	Number of unique reads	Total number of bases	Average raw coverage
Angus	109	82,263,951,806	8,137,666,488,753	25.74
Hereford	18	15,603,339,064	1,501,290,942,627	28.76
Limousin	12	3,704,169,818	357,264,463,240	10.27
Charolais	14	8,560,329,604	858,471,719,367	21.14
Simmental	11	8,902,705,282	885,698,817,042	27.76
Gelbvieh	8	6,366,906,096	633,479,558,830	27.31
Maine Anjou	5	4,061,220,172	403,867,224,031	27.85
Romagnola	4	901,544,762	89,666,842,589	7.73
Shorthorn	2	1,446,405,682	143,863,277,001	24.80
Red Angus	14	4,430,950,144	441,846,880,499	10.88
Holstein	55	13,650,662,246	1,358,163,462,700	8.52
Jersey	9	1,399,450,902	139,150,036,295	5.33
N’Dama	1	739,233,320	73,483,493,461	25.34
Brahman	11	1,871,667,422	167,772,161,118	5.26
Nelore	8	1,668,006,036	165,728,918,125	7.14
Gir	6	1,583,737,248	157,449,065,756	9.05
Beefmaster^a^	10	8,351,392,646	830,865,082,100	28.65
*Bison bison*	3	3,042,961,175	302,630,047,893	34.79
*Bubalus bubalis*	64	9,521,271,994	942,508,023,731	5.08

^a^Composite breed with an expected composition of 50 % Brahman, 25 % Hereford and 25 % Shorthorn

For the animals sequenced at MU, two independent sequencing libraries with average fragment sizes of 350 and 550-bp were constructed, and each one was independently sequenced. For, animals that were sequenced elsewhere, only a single library was constructed and sequenced. In general, 2 × 100-bp sequences were generated although the data were produced over a sufficiently long period so that Illumina Genome Analyzer, GAII and HiSeq2000/2500 instruments were used with all generations of Illumina sequencing chemistries.

RNA samples were sequenced from 98 tissues including the livers of 12 inbred Lewis (LEW/Crl) rats [3], an extensive series of tissues sampled from L1 Dominette 01449 and her daughter, a male calf, and from lung lesions, healthy lungs, bronchial lymph nodes, retropharyngeal lymph nodes, nasopharyngeal lymph nodes and pharyngeal tonsils collected at the peak of clinical disease from 43 Angus × Hereford cattle that were experimentally-challenged with bovine respiratory syncytial virus (BRSV), infectious bovine rhinotracheitis (IBR), bovine viral diarrhea virus (BVDV), *Mannheimia haemolytica*, *Pasteurella multocida* or *Mycoplasma bovis* [4, 18] [see Additional file 1: Table S1]. RNA-seq was also run on liver, small intestine, skeletal muscle, anterior pituitary and median eminence of the hypothalamus from 12 commercial Angus, from 6 to 12 Hereford and 12 Simmental × Angus animals that represented the phenotypic extremes of the distribution of residual feed intake, a measure of feed efficiency. For an additional 36 Angus animals that originated from the Circle A Ranch, Iberia, Missouri, RNA-seq was performed on liver samples and these animals were sampled to represent the extremes and center of the residual feed intake distribution. The remaining animals were Holsteins for which RNA-seq was performed on isolated neutrophils.

Preparation of the mRNA samples for sequencing was performed using the TruSeq RNA Sample Preparation Kit (Illumina, San Diego) and either 1 × 100-bp, 2 × 100-bp or 2 × 50-bp reads were obtained. Further details are in Chapple et al. [3] and Tizioto et al. [4].

### Processing of sequence reads

Raw sequence reads were first processed through FastQC (http://www.bioinformatics.babraham.ac.uk/projects/fastqc/) to obtain an initial assessment of quality and flag any potential issues. Then, exact duplicates of sequence reads were parsed into a separate file followed by adapter trimming using a custom Perl script that identified exact string matching to a user-supplied adapter sequence as described in Chapple et al. [3].

### Read preprocessing and alignment

Error correction was performed on DNA sequence reads using the QuorUM error correction algorithm [19]. Following error correction, all reads that were exact duplicates were again parsed into a separate file and reads that were shorter than 35 bases were also parsed into separate files. This process resulted in multiple files with different characteristics in terms of duplicate content and sequence read length that is a lossless process allowing files to be chosen for downstream analysis depending on the requirements of the analysis. Paired reads were aligned to the UMD3.1 bovine reference assembly using NextGENe 2.4.1 (SoftGenetics, LLC, State College), which required at least 35 contiguous bases with more than 95.0 % overall match, up to two allowable mismatched bases, and up to 100 allowable alignments of equal probability genome-wide.

For the RNA-seq data, TopHat v2.0.629 [20] was used to align the adapter trimmed reads to the *B. taurus* virtual transcriptome build and the UMD3.1 bovine reference genome by providing both a gene annotation file (NCBI *B. taurus* Annotation Release 103) and the reference genome assembly. TopHat first extracted transcript sequences and used Bowtie to align reads to the virtual transcriptome build. The reads that could not be mapped to the virtual transcriptome were next mapped to the genome assembly. These reads were converted to genomic mappings (spliced as necessary) and merged with the novel transcriptome mappings and junctions. Two mismatches and up to 3-bp indels were allowed in the alignment.

### Processing unmapped reads

Reads from DNA sequencing that remained unmapped after alignment to the reference genome were assembled using MaSuRCA 2.3.2 [21]. Reads from RNA sequencing that remained unmapped after alignment were assembled using Trinity version r20140717 [22, 23]. To maintain a paired read file structure, reads for which both the forward and reverse reads were unmapped or for which one read was unmapped while the other was mapped were collectively used for assembly.

Prior to pairwise alignment, contigs that were assembled from the unmapped DNA reads were sorted by size and only the contigs that were longer than 500 bases were aligned. Due to the smaller size of the RNA contigs, they were not filtered by size prior to pairwise alignment. Using the blast algorithm of BLAST + 2.2.30 [24], each DNA and RNA contig was aligned to the NCBI non-redundant nucleotide database and the most significant alignment was returned. The BLAST output was then parsed to determine the subject species, percent identity, length of match, number of mismatches, number of gaps, E-value, and overall score. Alignments were declared significant only if the alignment was longer than 200-bp for DNA or longer than 50-bp for RNA contigs. Only the best match for each aligned contig was reported. This output was summarized according to the total number of alignments per species, average and maximum percent identity, average and maximum length of match, and average E-value. For significant RNA alignments, the gene symbol corresponding to the GI (GenInfo Identifier) number for the alignment was identified when possible and recorded using the db2db tool in bioDBnet [25]. A unique list of gene symbols was constructed and the number of significant alignments to each gene was recorded.

### Variant calling and filtering

NextGENe 2.4.1 was used to call variants for the genomic data on an individual sample basis. Any position that differed from the reference assembly was considered as a putative variant and subject to downstream characterization and filtering. Putative variants were filtered based on several criteria such as coverage depth, allelic balance and forward/reverse read balance using custom Perl/SQL scripts. All variants were maintained in a custom PostgreSQL database (http://www.postgresql.org) where they were further characterized based on structural and functional annotations. A final genotype call set was generated, which required that each variant was observed in at least two individuals, was bi-allelic and passed all previous filtering criteria.

### Variant imputation, imputation accuracy and genome-wide association study

Data from Run4 of the 1000 Bull Genomes Project comprised whole-genome sequence (WGS) genotype calls for 35,431,201 variants on 1147 animals that covered 38 cattle breeds and had been phased and error-corrected using Beagle 4.0 [26]. These data were used to impute genotypes from 4010 registered Angus bulls to WGS in a two-step process. First, BovineSNP50 [27] (3769) genotypes were imputed to BovineHD (241) genotypes using Beagle 4.0 and second BovineHD genotypes were imputed to WGS using FImpute [28]. The Run4 dataset included 1,500,659 variants on bovine chromosome BTA7 (BTA for *B. taurus*), however, only 477,544 of these were variable in both the imputed data and in variants called at MU for 94 sequenced Angus bulls (included in Table 1) that were also represented in the set of the 4010 genotyped animals. These loci were used to assess the accuracy of imputation of BTA7 variants by calculating the correlations between imputed and sequence-called genotypes at each of the 477,544 variants for these 94 animals. After filtering variants to retain only those with a minor allele frequency (MAF) higher than 0.05, 9,430,182 imputed genome-wide variants and 397,241 variants on BTA7 remained. These were used to perform a genome-wide association study (GWAS) for birth weight using deregressed expected progeny differences (EPD) [29] and a custom developed genomic best linear unbiased prediction (BLUP) software, which included a genomic relationship matrix for all 4010 animals and sequentially fitted a regression on allele substitution effects for each imputed variant as a fixed effect in the model.

## Results

### Depth of coverage

**Fig. 1:**
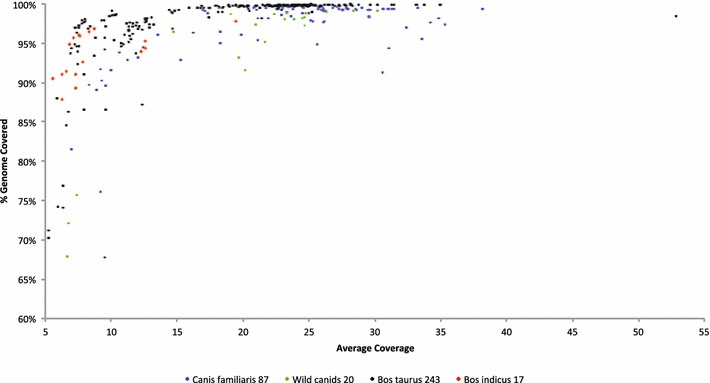
Percentage of bases in the reference assembly covered by at least one sequence read plotted against average genome coverage for each sample

Figure 1 shows the percentage of the reference genome that was covered by at least a single read for dog and cattle samples that had been sequenced to a depth between 4.8 and 52.8X. While this figure contains data for samples that had been sequenced from only a single library preparation and using different chemistry versions on both Illumina Genome Analyzer and HiSeq2000/2500 instruments, it is clear that samples that were sequenced to an average depth of <10X are much more variable in terms of the range of their reference genome coverage than were samples that were sequenced to an average depth of more than 10X. The 66 samples that were sequenced to an average depth of <10X had an average depth of reference genome coverage of 7.7X and, on average, covered only 89.7 ± 9.9 % of the reference genome with at least one read. Conversely, the 301 samples that were sequenced to an average depth of more than 10X had an average depth of reference genome coverage of 22.0X and, on average, covered 98.7 ± 1.8 % of the reference genome with at least one read. With an average depth of reference genome coverage of 24.2X, it was estimated that 99.5 % of the reference assembly was covered by at least one read.

**Fig. 2:**
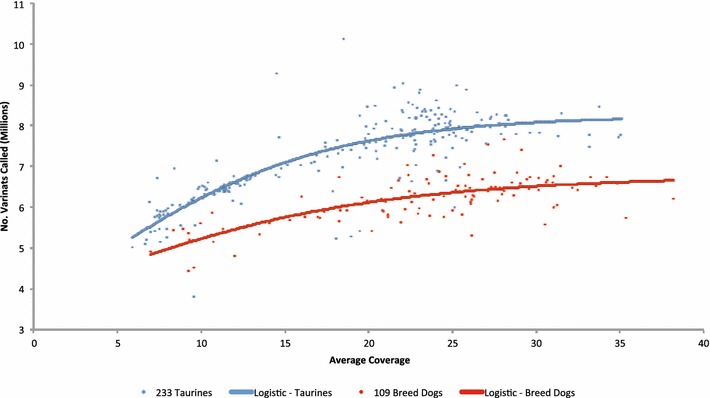
Number of variants called for each sample plotted against depth of reference assembly coverage. We analyzed 233 taurine cattle and 109 breed dogs for which the asymptotes of the logistic curves were at 8.247 and 6.767 million variants, respectively

Figure 2 shows the number of variants that were called for each sample plotted against average depth of reference genome coverage for 233 taurine cattle and 109 breed dogs. This figure also includes a logistic regression fit by ordinary least squares of the form:

$$\# {\text{variants }} = \frac{Asymptote}{{1 + e^{{ - \beta_{0} - \beta_{1} \times Depth}} }},$$

which was fit separately for dogs (*R*^2^ = 0.52; ${\hat{\beta }}_{0} = 0.206, {\hat{\beta }}_{1} = 0.102$) and cattle (*R*^2^ = 0.68; ${\hat{\beta }}_{0} = - 0.251, {\hat{\beta }}_{1} = 0.138$), where #variants is the number of variants detected in millions and *Depth* is the average depth of coverage of the reference genome for the sample. Asymptotes for taurine cattle and breed dogs were estimated to be equal to 8.247 and 6.767 million variants, respectively. Although the curves are not parallel, they suggest that about 95 % of the variation within the genome of a taurine individual is discovered at an average sequence depth of 23.2X and that of a breed dog at an average sequence depth of 26.9X. To detect 99 % of the variants present within the genome, the required average depths of sequence coverage of the reference genome were estimated to be 35.1 and 43.0X for cattle and dogs, respectively.

**Fig. 3:**
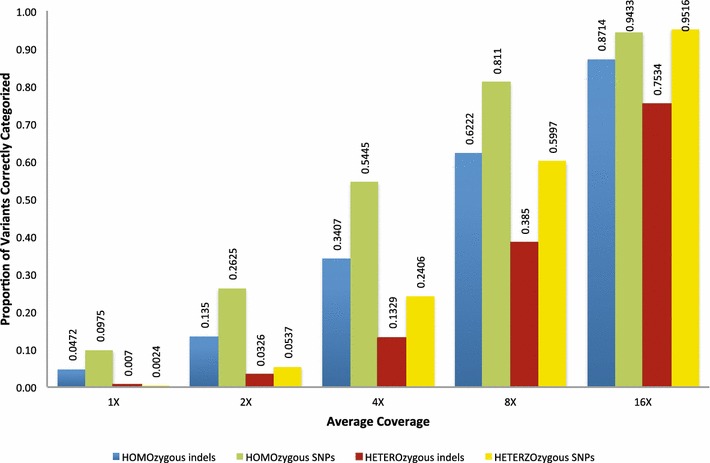
Proportion of variants called for each sample plotted against depth of reference assembly coverage. Ten replicate subsamples from one animal with a 23.3X sequence depth were sampled at depths of 1 to 16X and analyzed. Variants were called and classified into four classes that comprise homozygous or heterozygous indels or SNPs; the proportion of variants called is relative to the analysis of the 23.3X sequence data

Figure 3 shows the average proportion of variants called from the analysis of 10 replicate random subsamples from 1 to 16X sequence depth relative to the number of variants detected from the complete 23.3X reference genome coverage for an Angus bull. As expected, the detection of heterozygous sites is considerably more impacted than the detection of sites that are homozygous for an allele that is different to the reference genome allele at shallow depths of coverage. At an average depth of 8X, we detected only 62.2 % of the homozygous indels and 81.1 % of the SNPs homozygous for alleles differing from the reference allele that were found in the analysis of the 23.3X coverage data. These percentages decreased to 38.5 and 60 % for heterozygous indels and SNPs, respectively. Since the results in Fig. 2 suggest that, on average, only 95.6 % of the variants in the genome of this animal were detected in the analysis of the full 23.3X data, the percentage of homozygous and heterozygous sites discovered at the different sequence depths should be scaled by 0.956 to estimate the proportions present in the genome that were actually discovered.

### Alignment statistics

For the dogs and taurine cattle represented in Fig. 1 and the three bison individuals in Table 1, we aligned the sequences from all the canid individuals to the canFam3.1 reference assembly and from all of the bovid individuals to the UMD3.1 reference assembly. For the breed dogs, 94.12 % of the reads were mapped to canFam3.1 of which 85.59 % were perfect matches, whereas, for the wild dogs, 92.23 % of the reads were mapped to canFam3.1 of which 80.74 % were perfect matches. For the taurine individuals, 88.97 % of the reads were mapped to the UMD3.1 bovine reference, of which 79.44 % were perfect matches. However, when the sequences of the three bison individuals were aligned to the UMD3.1 bovine reference, 81.57 % of the reads mapped to it of which only 42.99 % were perfect matches.

### Identity of unmapped DNA and RNA sequencing reads

When the findings of this section were presented at the International Symposium on Animal Functional Genomics conference in Piacenza, they had not yet been published. While the analyses of the unmapped RNA-seq reads from the animals challenged with pathogens from the bovine respiratory disease complex are yet to be published, please refer to [2] for a more complete description of the work. However, here we present a synthesis of the work to draw the reader’s attention to several interesting findings including the metagenomic context of next-generation sequencing experiments, the occurrence of reference assemblies that are contaminated with sequences from other species and the ability to use the unmapped reads to identify sequences that are present in an individual that are not included in the reference assembly, which may be due either to errors in the reference assembly or insertions in the genome of the sequenced subject.

Based on a 52.8X average coverage of the reference genome by 2 × 100-bp reads produced from DNA extracted from white blood cells and liver tissue from L1 Dominette 01449, 7.2 % of the reads failed to align to the UMD3.1 bovine reference assembly. These reads were assembled into 69,230 contigs that ranged from 75 to 10,164 bp and represented 46.64 Mb of unique assembled sequence corresponding to 1.7 % of the reference genome [2]. The 42,086 contigs that were longer than 500 bp were aligned to the NCBI non-redundant nucleotide (nt) database and a significant alignment was found for approximately 51 % of them. The most common alignments were to *B. taurus* sequences. However, the second most common alignments were to *Onchocerca ochengi*, a nematode that is known to infect indicine cattle and has been extensively studied due to its similarity to the parasite *Onchocercavolvulus* that causes African River blindness in humans. We randomly sheared the *O. ochengi* reference genome to generate 2 × 100-bp paired-end sequence pseudo-reads with a random library DNA fragment size distribution between 200 and 500 bases (mean of 350 bases) by stepping through the genome in 1-bp steps. The same procedure was used to generate pseudo-reads for *Babesia bigemina* that was also detected in the contig search of the nt database, the sister species *O. volvulus* and five other species *Drosophila melanogaster*, *Caenorhabditis elegans*, *Echinococcus granulosus*, *Trichuris muris* and *Dictyocaulus viviparous* that were not found in the contig search of the nt database. By aligning the pseudo-reads for these species to the UMD3.1 reference assembly, we observed a 9.3–786.4-fold enrichment of alignments to *O. ochengi* relative to the other species and concluded that the *O. ochengi* assembly, based on samples obtained from cattle skin [30], is contaminated by bovine sequences. After removing the *O. ochengi* assembly alignments, 18,320 contigs were aligned to sequences from 132 species, of which 119 (90.2 %) were vertebrates with 17,306 (94.5 %) contigs. Fourteen contigs aligned to synthetic constructs, six to two plant species, six contigs to three strains of *Salmonella* and two viruses. Of considerably more interest, 988 (5.4 %) contigs aligned to the protist *B. bigemina* (1.04 %), and to the nematodes *Gongylonema pulchrum* (2.82 %), *Wuchereria bancrofti* (1.49 %), *Parascaris equorum* (0.04 %) and *Onchocerca flexuosa* (0.01 %).

An average of 6.0 % of the total of 577,753,827 RNA-seq reads remained unmapped across all of the 17 tissue samples (i.e., ~33,985,519) that were run as a single experiment at BGI. De novo assembly of these reads yielded a total of 43,961 contigs, with an average of 2586 contigs per tissue and an N50 (50 % of the assembled contigs are equal to or larger than this value) value of 321.6-bp. In total, the contigs spanned 14.8 Mb (~27.6 % of the transcribed genome assuming that it represents 2 % of the reference assembly) with an average of 871 kb per tissue. Of these, 35,632 (81.1 %) contigs returned alignments to 228 species. Again, these primarily represented vertebrates with 35,316 (99.1 %) contigs aligned to 112 (49.1 %) species. Three contigs aligned to synthetic constructs, two to two insect species, six to six plant species, two to two mold species, one to an algae and one to a yeast species. One hundred and twenty-six contigs aligned to 53 bacterial species, and 88 contigs aligned to 29 fungal species. Bovine herpesvirus was identified by 22 contigs and BRSV by a single contig. Seven nematode species including *G. pulchrum*, *O. flexuosa* and *W. bancrofti* were identified by 28 contigs. Finally, 12 protozoa species including *B. bigemina* were identified by 36 contigs.

By mapping the GI number of the most significant BLAST alignment to a gene symbol, we detected 17,856 contigs that aligned to 4412 *B. taurus* genes and 13,769 contigs that aligned to 4029 *B. bison bison*, *B. bubalis* or *Bos mutus* genes. We also aligned the RNA-seq contigs to the set of DNA contigs and found that ~21 % aligned with more than 98 % sequence identity. The RNA contigs spanned 1.2 Mb (2.6 %) of the 46.64 Mb represented by the DNA contigs.

For the 27 animals that had been challenged with optimized doses of pathogens responsible for bovine respiratory disease [4, 18], 6–16 % of the RNA-seq reads from lung lesions, healthy lungs, bronchial lymph nodes, retropharyngeal lymph nodes, nasopharyngeal lymph nodes and pharyngeal tonsils failed to align to the UMD3.1 bovine reference assembly. We pooled unmapped reads for each animal across tissues and again assembled these reads into contigs using Trinity and then queried the NCBI non-redundant nucleotide database to identify these contigs. Again most of the contigs aligned to vertebrate genes, which were highly enriched for immune function genes such as *beta*-*2*-*microglobulin* (*B2M*), *immunoglobulin lambda* (*IGL*), *immunoglobulin heavy chain* (*IGH*), *histocompatibility complex, class II, DQ beta, type 2* (*BOLA*-*DQB2*), *interleukin 3 receptor subunit alpha* (*IL3RA*), *immunoglobulin heavy constant gamma 1* (*IGHG1*), *leukocyte immunoglobulin*-*like receptor, A1, A2, A3 and A4* (*LILRA1*, *LILRA2*, *LILRA3* and *LILRA4*) and *integrin subunit alpha L* (*ITGAL*).

We also found that some of these contigs aligned to sequences from infectious pathogens, including some of the challenge pathogens, and others which are potentially new members of the bovine respiratory disease complex. We also found sequences representing many other organisms that are likely to be commensals. For one animal challenged with BRSV, we were able to build a significant portion of the virus’ genome sequence with two contigs of 9627 and 5544 bp that shared ~99 % sequence similarity to BRSV ATCC51908 (gi|17939982|gb|AF295543.1|AF295543). In addition, some contigs from the other animals that were challenged with BRSV also showed a high level of homology to both BRSV and the ovine respiratory syncytial virus M2 (~89 % sequence homology for a 969-bp contig). For the animals that were challenged with IBR, we consistently assembled contigs that were longer than 2800 bp with almost 100 % homology to bovine herpesvirus type 1.1. We also successfully predicted the presence of *M. bovis* in animals with significant lung consolidation (contigs with more than 2800 bp and ~99 % homology), whereas for animals without gross lesions, the contigs representing *M. bovis* were significantly smaller (≤300 bp), which suggested a much lower pathogen abundance. We did not detect any contigs that shared significant homology to *M. haemolytica* or BVDV in any of the challenged animals. We found contigs representing *M. bovis* and *Mycoplasma wenyonii* in one animal challenged with *M. haemolytica* and *Mycoplasma hyopneumoniae* in one animal challenged with BVDV. Additional species that were detected included: *Pseudomonas* sp., *Roseomonas* sp., *Janthinobacterium* sp., *Afipia* sp., *P. multocida*, *Histophilus somni*, *Bibersteinia trehalosi* (USDA-ARS-USMARC-190), *Chryseobacterium* sp., *Elizabethkingia anophelis*, *Actinoplanes missouriensis*, *Corynebacterium* sp., *Acinetobacter* sp., *M. hyopneumoniae* and *Lactobacillus* sp. which were consistently found with contigs that were up to 2026-bp long and with ~98 % sequence similarity.

### Imputation to WGS and GWAS suggests assembly errors

**Fig. 4:**
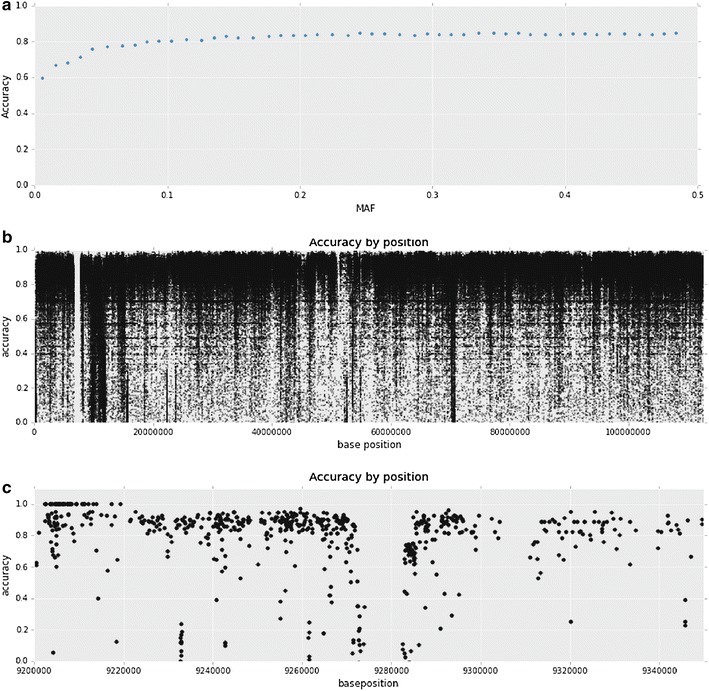
Accuracy of variants imputed to whole-genome sequence using Run4 of the 1000 Bull Genomes project data established by correlating imputed and independently sequence called genotypes on 94 registered Angus bulls. **a** By MAF and ignoring location on the chromosome. **b** Whole chromosome 7. **c** Chromosome 7 from 92 to 93.4 Mb

Imputation accuracies for 477,544 variants on BTA7 for 4010 registered Angus bulls are in Fig. 4a according to MAF. Although 800 K data were available for only 241 (6.0 %) of the 4010 genotyped animals, imputation accuracy reached at least 82 % for the common variants and fell to 60 % for the rarest variants only. Figure 4b shows the “Subterranean plot”, which provides imputation accuracy according to site on BTA7 and reveals several interesting features. First, there is a 1.07-Mb gap in the assembly of BTA7 between 6,722,059 and 7,796,216 bp on the UMD3.1 bovine assembly from the UCSC browser. Second, this figure reveals vertical bands (or deep “roots”) that represent regions of the chromosome where imputation accuracy is drastically reduced, presumably because the order of the SNPs provided by the reference assembly that was used to create the haplotypes for imputation is not correct. Figure 4b reveals that there are many such regions on BTA7 among which those between 9 and 12 Mb and around 17 and 72 Mb appear to be particularly problematic.

**Fig. 5:**
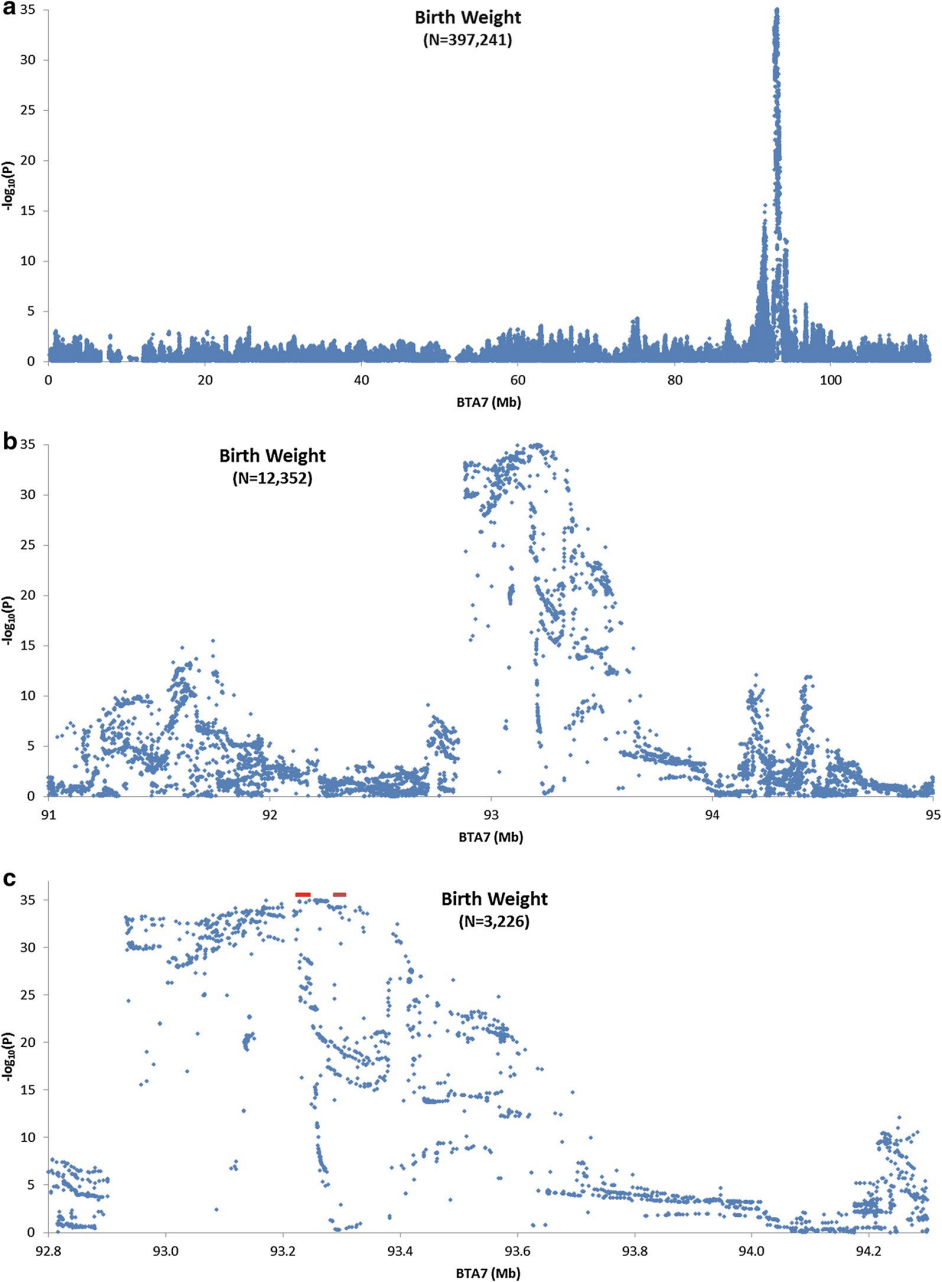
Association of SNPs imputed to sequence level with birth weight for 3570 registered Angus bulls. **a** Associations for 397,241 SNPs on chromosome 7. **b** Associations for 12,352 SNPs between 91 and 95 Mb on chromosome 7. **c** Associations for 3226 SNPs between 92.8 and 94.2 Mb on chromosome 7. *Red* and *purple bars* represent *LOC101905238* and *ARRDC3*, respectively

The imputed data were filtered to retain 397,241 variants with a MAF higher than 0.05 and a GWAS was performed on 3505 bulls with deregressed estimates of breeding values for birth weight. Figure 5a shows that a very large quantitative trait locus (QTL) at about 93 Mb on BTA7 was detected and Fig. 5b, c each provide a zoom in to show the regions between 91 and 95 Mb (that includes 12,352 imputed variants) and between 92.8 and 94.2 Mb (3226 imputed variants), respectively. Both Fig. 5b, c reveal a discontinuity in the GWAS signal at 92.8 Mb, which is illustrated by a rapid decline in −log_10_P values as the plotted data points move from the QTL towards the centromere. Figure 4c shows that there is a gap in the evaluated accuracy of the SNP genotypes in this region and that imputation accuracy falls to nearly 0 on each side of the gap, which clearly suggests that the reference assembly in this region lacks some sequence. Figure 5c also shows the locations of the two annotated loci that are located closest to the peak QTL signal. The red and purple bars represent *LOC101905238* and *ARRDC3*, respectively and neither of these appears to carry the mutation that is responsible for the QTL, which suggests that the causal variant may lie within a regulatory element.

## Discussion

### Depth of coverage

The depth to which the genome of an individual is sequenced affects both breadth of genome coverage and number of variants that are identified [31]. For the sequencing of the genome of dogs with neurological disorders that are postulated to be inherited as autosomal recessive Mendelian loss-of-function mutations, rather than deep-sequencing trios as was effectively done for human disorders [32], we chose to sequence only the affected individuals and to compare the variants that were detected in one or more cases to those found in control animals, which generally belonged to other breeds and were afflicted with different disorders. Provided that control individuals are from breeds that do not segregate for the disease, we can reasonably safely (but are not required to) assume that all the controls will be homozygous for the reference allele at the causal variant. It has been reported that this strategy works reasonably well [6–13] but it requires that animals are sequenced to a sufficient depth for heterozygous variants to be correctly identified across most of the genome. Figures 1, 2 and 3 jointly suggest that the targeted sequence depth should be at least 30X for exhaustive and precise genome-wide genotyping-by-sequencing of individual animals. In particular, we note that the proportion of the reference genome that remains uncovered by a single read is non negligible (e.g., 5–10 %) even when the genome is sequenced to an average depth of 5–10X (Fig. 1). This is clearly more than would be expected based on the Poisson distribution, which indicates that systematic biases remain within Illumina’s sequencing chemistry; however, this has improved substantially over time and the data in Fig. 1 represent samples that were sequenced using either one or two libraries and with different versions of sequencing chemistry.

Applications of genotyping-by-sequencing in animal breeding seem to go in two directions. Because the effective population sizes of most breeds of cattle are only about 100 [33], relatively few genomes need to be accurately genotyped and phased to recover most of the haplotypes that are present within each breed. Furthermore, phase relationships are preserved among breeds for distances up to 10 kb [34], which indicates that data on individuals from different breeds may be useful to predict haplotypes and genotypes of animals from other breeds; thus it may not be necessary to deep sequence 100–200 individuals from each breed to capture the haplotypic diversity within cattle. Imputation from the BovineHD SNP set for BTA1 to WGS in Holstein cattle using 114 sequenced Holstein bulls produced an accuracy of imputed genotypes of 83 % [35]. Using a combined bovine dairy breed reference population yielded the highest accuracy of imputation of BTA29 BovineHD SNP genotypes to WGS in a test population comprising three dairy breeds [36]. These approaches for imputing genotypes do not involve the use of pedigree information but do require that the animals are first genotyped with a high-density SNP array. The second approach for imputing genotypes to WGS requires that all individuals within a population are sequenced at shallow depth, that a low-density genotyping assay is used to detect the genomic regions that are identical by descent among the individuals and that the sequence of these regions is accurately inferred by using the sequence data from individuals that share common haplotypes [37]. It seems clear that these approaches will become much more common in programs for the genetic improvement of livestock and that the data in Figs. 1, 2 and 3 will guide the design of sequencing experiments regarding the total sequence depth that is required to accurately estimate haplotype structure.

In Fig. 2, the asymptotes for taurine cattle and breed dogs were estimated to equal 8.247 million and 6.767 million variants, respectively. Rather than suggesting that the genomic variation in dogs represents only about 82.1 % of that in taurine cattle, it is most likely that variant calling is significantly influenced by the shape of the phylogeny, position of the reference individual within the phylogeny and the nature of the sample of sequenced individuals for each species. For example, if the phylogeny of a species included a number of very closely-related breeds and one very distantly-related breed, the selection of a member from the distantly-related breed as the reference genome individual would result in a larger average number of variants being detected in a random sample of individuals from all of the breeds than if a member of one of the closely-related breeds was selected as the reference genome individual. Likewise, our samples of sequenced individuals were not randomly selected from various breeds, and in particular, almost 50 % of the sequenced taurine cattle belonged to the Angus breed, which is closely related to the Hereford breed [38]. However, for the 17 registered Hereford bulls that we sequenced to an average depth of 22.4X (excluding the reference animal), we detected an average of 6.709 million variants relative to the reference Hereford genome, which is almost as much as that represented by the diversity in the 61 sequenced dog breeds when compared to the reference Boxer breed. We sequenced only one Boxer dog to an average depth of 28.1X and found only 4.108 million variants. Together, these data strongly support that there is more diversity within domesticated taurine cattle than within all the breed dogs.

When Angus, Holstein and Hanwoo bulls were sequenced to an average depth of 22, 19 and 45.6X using an ABI SOLiD sequencer, only 3.7 million, 3.2 million and 4.7 million SNPs were reported [39, 40]. While our data represent both SNPs and small indels (<10 % of the total variants detected), here we clearly report considerably larger numbers of variants per individual than those found in previous studies. The reasons for this come down to the objectives of the analysis and how the variant calls were filtered. While many studies aim at filtering out as many false positives as possible, which also removes some true positives, to define a set of variants with a low error rate [39, 40], our objective was to filter less stringently so that among the bovine individuals analyzed, we would be able to identify those that carried rare variants, some of which were likely to be recessive embryonic lethal, to be included in the design of the GGP-F250 assay. Consequently our data contain more true and false positives and are not directly comparable to the previously published results of Stothard et al. [39] or Lee et al. [40].

An average number of 8.301 million variants were detected for 15 wolves sequenced to an average depth of 19.2X, which is about 36.8 % more than for breed dogs sequenced to the same depth. However, we found an average of 12.248 million variants for 26 indicine and taurine × indicine crossbred animals sequenced to an average depth of 12.1X, or about 84.5 % more variants than were found for taurine cattle sequenced to the same average depth. Thus, the average indicine animal appears to possess about 1.85 more diversity than the average taurine animal, which is slightly less than the value of 2.5X, which was estimated based on nucleotide differences per chromosome by the Bovine HapMap Consortium [34]. These results are consistent with the evolutionary histories of both species since dogs were domesticated from wolves approximately 15,000 years ago [1] and taurine and indicine cattle diverged at least 200,000 years ago [41].

### Alignment statistics

We found that a much larger percentage of sequence reads mapped to the canFam3.1 dog reference assembly for an average breed dog than that to the UMD3.1 cow reference assembly for an average taurine individual. This suggests that two randomly sampled dogs are more similar to each other than two randomly sampled taurine individuals, or that the quality of the dog reference assembly is much higher than that of the cow reference assembly, or perhaps both. We have already provided strong evidence that there is greater diversity among cattle than among dogs, which is no doubt due to the founder events and much stronger bottlenecks that occurred during the formation of dog breeds than during the formation of bovine breeds. In itself, this indicates that the average breed dog is more similar to another breed dog than one taurine cow to another. While we have not attempted to directly compare the quality of the two reference genome assemblies, our analyses in the following sections indicate that there are numerous errors in the UMD3.1 bovine assembly and we speculate that the difference in alignment metrics is because dogs are both more similar to each other genetically than are cattle and also because the dog reference assembly contains fewer errors than the UMD3.1 bovine assembly.

The alignment statistics also show strong phylogenetic signals and that wolves are more similar to breed dogs than bison to cattle. Again, dogs were domesticated from wolves approximately 15,000 years ago while North American bison and cattle diverged at least 1 Myr ago [38, 42].

### Identity of unmapped DNA and RNA sequence reads

When paired-end short read DNA or RNA sequences are aligned to a reference assembly, typically about 10 % of the reads fail to align even if mismatches and small indels are allowed in the mapping process, these being due to sequence divergence between the reference and sequenced individuals and to sequencing errors. Sequences that are actually present in the reference assembly can be at least partially recovered using alignment tools that account for base quality scores, provided that they are not too divergent [43]. However, the unmapped reads are generally not considered further in the analysis and they are typically thought to contain reads that are more divergent from the reference than allowed, reads that contain motifs that are repeated throughout the genome and reads that contain sequences that are misassembled or missing from the reference genome. However, until recently [44, 45], there was little interest in examining this issue and characterizing the relative importance of the contributing factors. We were fortunate to have available DNA and RNA sequence datasets that were derived from tissues from L1 Dominette 01449, the same individual that was used to create the Sanger-based reference genome assembly for cow. Consequently, we were able to remove the issue of sequence divergence from the mapping process and to reduce the percentage of unmapped reads to 7.2 % for DNA sequences and 6.0 % for RNA sequences. By assembling these reads and querying them against the NCBI nucleotide database, we found that the vast majority (94.5 % for DNA and 99.1 % for RNA contigs) of the contigs assembled from the unmapped reads represented vertebrate sequences. By mapping the GI number from the most significant BLAST alignment of each RNA contig to a gene symbol, we estimated that 4412 annotated bovine genes were represented in the 17,856 alignments to *B. taurus* sequences. Of the 13,769 significant alignments to bison, water buffalo or yak, 4029 genes were represented and only one gene was in common with the 4412 genes detected from the alignments to *B. taurus* sequences. This suggests that 8440 unique genes or as much as 42 % of the bovine coding genome is misassembled. A list of the affected genes is in Whitacre et al. [2].

We also found that a large number of the alignments represented by small numbers of contigs were with sequences from many fungal, bacterial, insect, plant, mold, algae and yeast species. The small number of the represented contigs suggests that either they are type I errors or they may represent environmental contaminants that occurred during tissue sampling or during nucleic acid preparation. Of greater interest was the relatively large number of contigs that mapped to protozoa and nematode species, which clearly indicate the presence of parasites infecting the reference animal. As expected, we found an enrichment of contigs representing *B. bigemina*, a blood borne parasite, assembled from the unmapped sequences derived from DNA extracted from white blood cells and contigs representing the nematodes *G. pulchrum*, *W. bancrofti*, *P. equorum* and *O. flexuosa* assembled from the unmapped sequences derived from DNA extracted from liver cells. Therefore, while the majority of the unmapped reads identified regions of the reference genome that are misassembled or missing, they also contain metagenomic information that is indicative of parasites, pathogens and commensals living in the tissues from which the nucleic acids were extracted. In addition to the recovery of divergent genomic regions between pea aphid biotypes, Gouin et al. [45] also found sequences from symbionts in the unmapped reads.

Genomic DNA sequencing seemed to be more powerful than RNA sequencing to identify the members of the sequenced community possibly due to an inherent, but unidentified, selection for vertebrate RNA in the creation of the sequencing libraries or possibly a large bias towards host tissue gene expression if, for example, the parasites exist as eggs with limited gene expression. Finally, the identification of species by mapping contigs to the NCBI nucleotide database simply results in the identification of the most similar sequence for a sequenced species and the vast majority of species on the planet have yet to be identified let alone sequenced. For example, *B. bigemina* is a tick-borne parasite that is primarily found in tropical and subtropical regions of the world and causes significant morbidity and mortality in cattle. *B. bigemina* is particularly prevalent in Asia, Africa, Central and South America, parts of southern Europe, and Australia. *B. bigemina* and its vector were formerly enzootic throughout much of the southern United States, but now are found only in a quarantine buffer zone along the Mexican border [46]. L1 Dominette 01449 lived her entire life in Miles City, Montana, USA and was therefore almost certainly not infected by *B. bigemina*. However, she was infected by a related protozoal species that may not yet be identified, let alone sequenced.

Analysis of unmapped reads from the animals challenged with pathogens responsible for bovine respiratory disease again revealed a large number of genes that are either misassembled, partially represented or completely missing from the reference assembly. Genes with immune system functions were, of course, overrepresented in these unmapped reads since these were stimulated in the challenge study and we have not yet determined the extent to which these are represented in the analysis of data from L1 Dominette 01449. However, we also assembled contigs representing infectious pathogens (including some of the challenge pathogens), potentially new pathogens which may be related to the bovine respiratory disease complex, and other organisms that are likely to be commensals. Some of these organisms such as *B. trehalosi, H. somni, Pseudomas* sp. and *P. multocida* are well known to cause respiratory diseases [47–50]. These organisms colonize the respiratory tract after taking advantage of the immunosuppression caused by the challenge pathogen. These missing or partially missing genes influence our ability to detect coding variants in WGS projects, to detect genes that are differentially expressed, particularly if completely missing from the reference assembly, and to detect isoforms, which is of considerable concern to studies examining the regulation of genes involved in the immune response.

### Imputation to WGS and GWAS suggest assembly errors

In spite of a relatively small number of animals genotyped with the BovineHD assay, we were able to achieve imputation accuracies in registered cattle that were comparable to those achieved in Holstein cattle [35] and a mixed population of dairy breeds [36], which is probably due to the larger training set of sequenced animals provided by the 1000 Bulls Project used here. However, rather than use masked variants for the computation of the accuracy of imputation, we used a set of independently sequenced animals with variants called using a completely independent analysis platform. Considering that these animals were sequenced to a sequence depth allowing the identification of about 95 % of the variants in their genomes and that we took no steps to phase and correct for errors the genotype calls for these bulls, this source of error should result in an underestimation of the accuracy of WGS imputation.

While the 1.07-Mb gap between 6,722,059 and 7,796,216-bp on BTA7 is annotated in the UMD3.1 bovine assembly, it is unclear how a gap of this size could have been inserted into the reference assembly in the first place. However, there are no large gaps annotated in the UMD3.1 assembly of BTA7 at 92.8 Mb but the Subterranean plot (Fig. 4c) reveals that there are no imputed SNPs for which imputation accuracy could be estimated in this region, which strongly suggests that the region is misassembled. Figure 5b, c suggest that this potential assembly error does not affect the precise localization of the large effect QTL at 93.25 Mb, but we cannot be certain that this is the case depending on the specific nature of the misassembly.

Based on the analysis of the 50 K data, the QTL located at 93.25 Mb was previously found to affect all stature, size and growth-related traits and to be the QTL with the largest effect discovered in the analysis of 17 traits recorded in Angus cattle i.e., it explained 7 % of the additive genetic variance in birth weight [33]. This QTL affects all growth, stature and size-related traits including calving ease and is also known to segregate and affect growth and feed efficiency traits in other breeds [51, 52]. The identification of the causal variant underlying this QTL would considerably help to improve the accuracy of estimated breeding values for all the traits that it affects and the proximity of the *arrestin domain containing 3* (*ARRDC3*) gene that encodes a major regulator of growth suggests that it is a suitable candidate gene. Expression of *ARRDC3* is down-regulated in breast cancers compared to normal tissue, and expression decreases with tumor grade, metastases, and recurrences. Conversely, the over-expression of *ARRDC3* in MDA-MB-231 basal-like breast cancer cells represses cell proliferation, migration, invasion, growth in soft agar, and tumorigenicity following injection in nude mice [53]. The location of the peak QTL signal in Fig. 5c suggests that the causal mutation may lie in an element that regulates the ubiquitous expression of *ARRDC3* in all tissues and specifically causes the gene to be down-regulated in fast-growing animals. Since we have performed RNA-seq experiments on the livers of 38 Angus steers that were also genotyped with the BovineSNP50 assay, the genotypes at the QTL for these animals could be determined based on the SNP data and the liver expression of *ARRDC3* could be compared across the genotype groups to test this hypothesis. Also, an important future step will be to functionally characterize the regulatory regions that are present in DNA derived from a tissue such as liver to refine the genomic regions that are candidates for harboring the QTL. Because we have already identified most of the common variants that are present in these regions from the WGS analysis, it should be a relatively straightforward process to genotype or impute these variants into multiple populations representing different breeds and identify a relatively small number of candidate mutations by meta-analysis across breeds and populations.

## Conclusions

We are at an interesting point in the history of livestock genomics where we know the sequence context of the majority of the common variants that are present within the genome of a species, but with the exception of variants that occur in the protein-coding regions of the genome, we know very little about the potential for functionality of much of the variation. Therefore, a clear direction for future infrastructural development is the improvement of the reference assemblies to remove existing assembly errors and include sequences that are currently missing. In parallel, there is a dire need to begin the process of annotating the regulatory regions of the genome that clearly underlie many of the QTL regions detected to date. The Functional Annotation of Animal Genomes (FAANG) project [54] seeks to annotate functional elements, but should first address the limitations in assembly quality. With the support of the USDA ARS, USDA NIFA AFRI and the NRSP8 Cattle Coordinators, a 70X Pac-Bio assembly for the genome of L1 Dominette 01449 is currently under development and is expected to result in a significantly improved reference assembly in 2016.

Genotyping-by-sequencing and the use of WGS imputation will clearly become more important for livestock improvement in the future. Low coverage sequencing of most of the members within a population will allow the accurate imputation to WGS of these individuals using pooled sequences for regions of the genome that are identical-by-descent between individuals, although the tools to accomplish this have yet to be developed. In conjunction, this will allow the identification of candidate causal variants for QTL genome-wide as well as those with consistent directions of allelic effects on phenotypes and highly correlated phenotypes across populations (with some assumptions regarding epistasis and genotype × environment interactions). These discoveries will be of great importance for the improvement of traits such as disease resistance and feed efficiency, which are not routinely phenotyped in many livestock populations.

## Availability of data and material

All data generated at the University of Missouri will become publicly available at the time of publication. See the relevant publication cited in the manuscript to obtain the appropriate accession numbers and data repository. Perl and SQL scripts used for variant filtering are available from RDS on request. GBLUP software is available from JFT on request.

## Additional file

10.1186/s12711-016-0237-6 Description of tissue samples analyzed by RNA-seq by species or breed and the project associated with the analysis.
